# Feasibility of high-throughput sequencing in clinical routine cancer care: lessons from the cancer pilot project of the France Genomic Medicine 2025 plan

**DOI:** 10.1136/esmoopen-2020-000744

**Published:** 2020-07-26

**Authors:** Céline Auzanneau, Delphine Bacq, Carine Bellera, Hélène Blons, Anne Boland, Marlène Boucheix, Aurélien Bourdon, Emmanuelle Chollet, Christine Chomienne, Jean-François Deleuze, Christelle Delmas, Derek Dinart, Hélène Espérou, Flore Geillon, Damien Geneste, Antoine Italiano, Delphine Jean, Emmanuel Khalifa, Yec'han Laizet, Pierre Laurent-Puig, Franck Lethimonnier, Claire Lévy-Marchal, Carlo Lucchesi, Carine Malle, Pierre Mancini, Simone Mathoulin-Pélissier, Vincent Meyer, Palomares Marie-Ange, Géraldine Perkins, Sabrina Sellan-Albert, Isabelle Soubeyran, Cédric Wallet

**Affiliations:** 1 Unité de pathologie moléculaire, Institut Bergonié, Bordeaux, France; 2 U1218, Institut Bergonié, Institut national de la santé et de la recherche médicale, Bordeaux, France; 3 Centre national de recherche en génétique humaine, Institut de biologie François-Jacob, Commissariat à l’énergie atomique et aux énergies alternatives, Evry, France; 4 Institut de santé publique, d'épidémiologie et de développement, Université de Bordeaux, Bordeaux, France; 5 CIC-EC1401/EUCLID, Institut national de la santé et de la recherche médicale, Bordeaux, France; 6 Service de pharmacogénétique et d'oncologie moléculaire, Hopital Europeen Georges Pompidou, Paris, France; 7 U1147, Centre universitaire des Saint-Pères, Institut national de la santé et de la recherche médicale, Paris, France; 8 Unité de bioinformatique, Institut Bergonié, Bordeaux, France; 9 ITMO Cancer, Alliance nationale pour les sciences de la vie et de la santé, Paris, France; 10 Institut National du Cancer, Boulogne-Billancourt, France; 11 Centre de référence, d'innovation et d'expertise, US39, Commissariat à l’énergie atomique et aux énergies alternatives, Evry, France; 12 Institut de santé publique, Pôle recherche clinique, Institut national de la santé et de la recherche médicale, Paris, France; 13 Fédération francophone de cancérologie digestive, Dijon, France; 14 Unités Essais cliniques de phase précoce et Sarcomes, Institut Bergonié, Bordeaux, France; 15 Service de génétique médicale et clinique, Hopital Europeen Georges Pompidou, Paris, France; 16 ITMO Technologies pour la santé, Alliance nationale pour les sciences de la vie et de la santé, Paris, France; 17 Service de génétique médicale et clinique, HEGP, Paris, Île-de-France, France

**Keywords:** WES/RNA sequencing, next-generation sequencing, targeted therapy, clinical practice, guidelines

## Abstract

**Background:**

Whole exome sequencing and RNA sequencing (WES/RNASeq) should now be implemented in the clinical practice in order to increase access to optimal care for cancer patients. Providing results to Tumour Boards in a relevant time frame—that is, compatible with the clinical pathway—is crucial. Assessing the feasibility of this implementation in the French care system is the primary objective of the Multipli study, as one of the four pilot projects of the national France Genomic Medicine 2025 (FGM 2025) plan. The Multipli study encompasses two innovative trials which will be driven in around 2400 patients suffering from a soft-tissue sarcoma (Multisarc) or a metastatic colorectal carcinoma (Acompli).

**Methods:**

Prior to launching the FGM 2025 cancer pilot study itself, the performance of the Multipli genomic workflow has been evaluated through each step, from the samples collection to the Molecular Tumour Board (MTB) report. Two Multipli-assigned INCa-labelled molecular genetics centres, the CEA-CNRGH sequencing platform and the Institut Bergonié’s Bioinformatics Platform were involved in a multicentric study. The duration of each step of the genomic workflow was monitored and bottlenecks were identified.

**Results:**

Thirty barriers which could affect the quality of the samples, sequencing results and the duration of each step of the genomic pathway were identified and mastered. The global turnaround time from the sample reception to the MTB report was of 44 calendar days.

**Conclusion:**

Our results demonstrate the feasibility of tumour genomic analysis by WES/RNASeq within a time frame compatible with the current cancer patient care. Lessons learnt from the Multipli WES/RNASeq Platforms Workflow Study will constitute guidelines for the forthcoming Multipli study and more broadly for the future clinical routine practice in the first two France Genomic Medicine 2025 platforms.

**Key questions**

**What is already known about this subject?**

- Identification of molecular abnormalities in cancer has improved cancer knowledge and patient care.
- Currently, these molecular abnormalities are identified by next-generation sequencing panels with a limited number of known candidate gene sequences to adapt treatment after first-line therapy.
- High-throughput technologies such as whole exome sequencing and RNA sequencing (WES/RNASeq) provide the possibility to explore more extensively the tumour genome and find out new genomic alterations to be targeted.

**What does this study add?**

- This study provides an example of a workflow for implementing WES/RNASeq in cancer care in a national multisite context.
- This study identifies and provides answers for 30 barriers that could affect the quality and turnaround time of WES/RNASeq in a routine setting.

**How might this impact on clinical practice?**

- Procedures constitute guidelines both for the French Multipli study and more broadly for the future French clinical cancer care routine.
- This study suggests standard operating procedures for WES/RNASeq in cancer clinical care that provide timely molecular characterisation after first-line therapy.

## Background

The benefits of targeted molecular analysis of tumour genomes have now been demonstrated for diagnostic, prognostic and theranostic purposes. Under the auspices of INCa (the French National Cancer Institute), 28 molecular genetics centres have been set across the country to perform molecular tests on tumour material in routine practice, using targeted next-generation sequencing (NGS) techniques.

High-throughput sequencing techniques open up the possibility of an even more extensive study of tumour genomic landscapes, which will help to make progress in the deciphering of cancer biology, the classification of tumours^1 2^ and the identification of new genomic alterations to be targeted.^3 4^ This extensive approach is expected to be more broadly implemented in the clinical cancer care routine, allowing precision cancer medicine to be developed and becoming a reality. As a next step to be considered, a growing number of studies explore the conditions under which implementation of high-throughput whole exome sequencing and RNA sequencing (WES/RNASeq) in the clinics will be valid in terms of feasibility, results accuracy, clinical benefit, and so on.^5–12^ Among these conditions, analysis of the patient’s tumour genomic characteristics has to be achieved in a time frame compatible with the patient care. Assessing the feasibility of this major issue in the French clinical context (see online supplementary data section, figure SD1) is the primary objective of the Multipli study, as part of the national France Genomic Medicine 2025 (FGM 2025) plan (detailed in the online supplementary data section, figure SD2).^13–15^

10.1136/esmoopen-2020-000744.supp1 Supplementary data

The FGM 2025 plan, launched in 2016, aims to construct a French medical and industrial system to introduce precision medicine into the routine care pathway. The plan is articulated around 3 main objectives and 14 measures. Among those, measure 5 aims to detect and overcome the technological, clinical and regulatory obstacles encountered along the genomic care pathway with respect to three broad groups of diseases and to a sample of the general population. Four pilot projects have been designed for this purpose, including the Multipli project for cancer disease. A preliminary multicentre study—the Multipli WES/RNASeq Platforms Workflow Study—was conducted to refine the Multipli genomic pipeline settings.

The aim of the present study was to evaluate the throughput and turnaround time across the pathway from sample reception to the Molecular Tumour Board (MTB) report based on a multicentre study and two WES/RNASeq Platforms.

## Methods

The Multipli WES/RNASeq Platforms Workflow Study was conducted on banked blood and tumour samples from metastatic colorectal cancer (CRC) patients or advanced/metastatic soft tissue sarcoma (STS) patients either in the care setting (Hopital Europeen Georges Pompidou (HEGP), Paris) or as they were enrolled in the BIP (Bergonié Institute Profilage) clinical trial (Institut Bergonié, Bordeaux). Patients were requested to give their agreement to the re-use of their samples according to French regulations. As exome sequencing of tumours requires parallel constitutional DNA sequencing, patients were also informed of the possibility of secondary findings, that is, identification of germline known pathogenic or expected pathogenic variants not related to their primary diagnosis. Their wish to have or not a return on these information was recorded.

**Figure 1:**
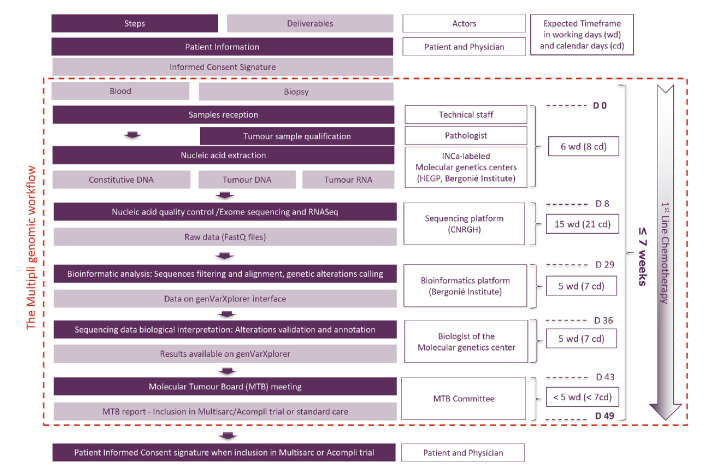
The Multipli WES/RNASeq Platforms Workflow Study assessed in situ the genomic workflow of the forthcoming Multipli study. Steps, deliverables, actors and expected time frames are shown. The evaluation study was designed to check step by step the performance of the Multipli genomic workflow, that is, that valid MTB reports can be obtained no later than 7 weeks (49 calendar days, 35 working days) after the samples’ reception at an INCa-labelled molecular genetics centre. D 0, Day 0; WES/RNASeq, whole exome sequencing and RNA sequencing.

Subsequent to informed consent, a patient identification number (patient ID) was assigned to the patient’s samples (blood and tumour). Three samples per patient (one blood sample for constitutional DNA extraction and two tumour samples for tumour DNA and tumour RNA extraction) were sent to one of the two Multipli-assigned INCa-labelled molecular genetics centres (HEGP or Institut Bergonié). Day 0 was defined as the day the samples were received at the Multipli genomic WES/RNASeq Platforms. From there onwards turnaround times were monitored as described in the workflow assessment diagram (figure 1). Five major steps had been previously defined in the process with definition of expected times. The actual time for each sample were precisely recorded.

Sequencing was performed at the CNRGH sequencing platform as detailed in the *online supplementary data section* (*‘*Nucleic acids qualification’ *and ‘*Sequencing parameters’ *subsection*s). Bioinformatic analysis was conducted at the Multipli-assigned bioinformatics platform of the Institut Bergonié. Computer supported biological interpretation was performed through genVarXplorer, a tool developed by the bioinformatics platform of the Institut Bergonié, as described in the *online supplementary data section* (*‘*Bioinformatics’ *and ‘*Biological’ *subsections*).

## Results and discussion

**Figure 2:**
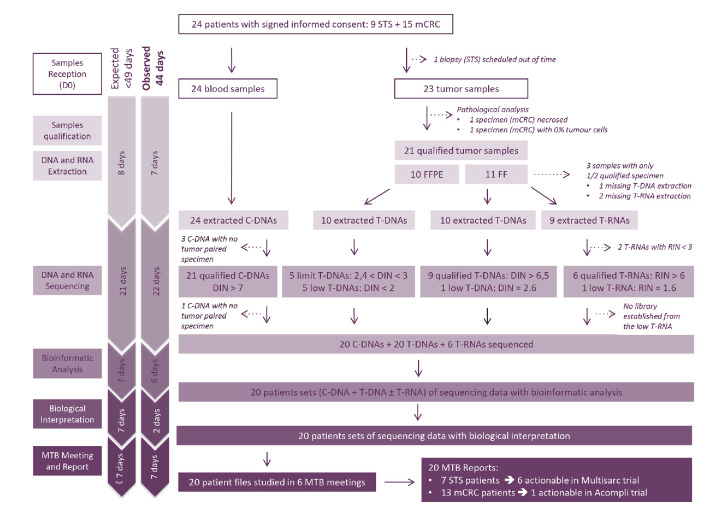
Overview of the Multipli WES/RNASeq Platforms Workflow Study. C-DNA, constitutional DNA; D 0, Day 0; mCRC, metastatic colorectal carcinoma; MTB, Molecular Tumour Board; STS, soft tissue sarcoma; T-DNA, tumour DNA; T-RNA, tumour RNA; WES/RNASeq, whole exome sequencing and RNA sequencing.

From July to August 2017, 24 patients—9 with an advanced STS, and 15 with a metastatic colorectal carcinoma (mCRC)—were included in the evaluation study (figure 2). Details are presented in the *online supplementary data section* (*‘*Overview of the study’ *subsection*).

**Figure 3:**
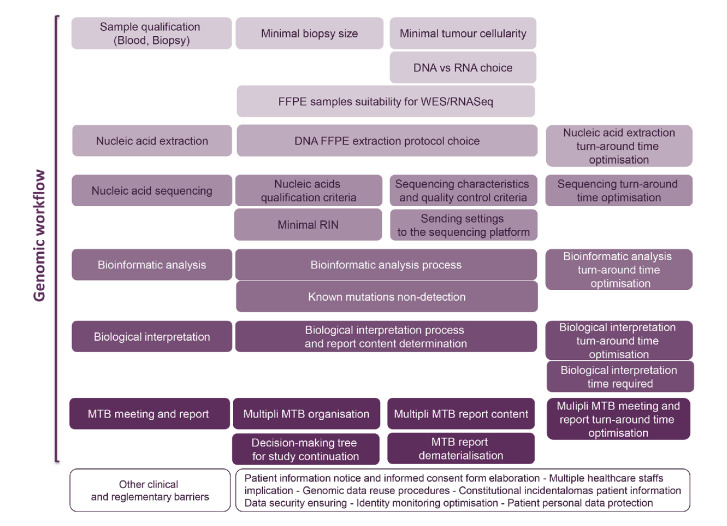
Barriers identified and mastered in the Multipli WES/RNASeq Platforms Workflow Study. FFPE, Formalin-Fixed Paraffin-Embedded; MTB, Molecular Tumour Board; RIN, RNA Integrity Number; WES/RNASeq, whole exome sequencing and RNA sequencing.

Thirty barriers potentially impacting the feasibility of high-throughput sequencing in clinical care were identified and mastered (figure 3). The main identified barriers related to technological aspects of the genomic workflow duration, DNA and RNA integrity as well as management and report delivery are detailed below. Additional information such as the issue of Formalin-Fixed Paraffin-Embedded (FFPE) stored samples, nucleic acid quality criteria, bioinformatics and Tumour Board interpretation are presented in the *online supplementary data section*.

### Turnaround times

The global turnaround time observed was of 44 calendar days from the sample reception to the MTB report. It fulfils the duration of 7 weeks expected to provide tumour genomic analysis results to the clinician in a time frame compatible with the current patient care. The evaluation study identified several key points concerning sample shipment that have to be strictly respected to avoid time wasting at the sequencing platform. It is necessary to notify nucleic acid quantities and patient gender, and to send sample pairs (tumour and blood) simultaneously. Samples should be sent 1 day a week, by Wednesday at best in order to allow the sequencing platform to process them by Friday, thus avoiding the samples to remain in transit during the weekend. Given that samples will not be processed on site in the Multipli study, rules for shipment and for data transfer between remote sites through dedicated protected servers are particularly important.

### Biopsy characteristics

Predefined tumour biopsy characteristics were evaluated during the study, which identified the following prerequisites. Collection of tumour biopsies guided by ultrasound or CT scan need to be obtained with a 18 gauge needle to provide cores of at least 20 nm in length. A minimum of 3 cores must be collected in order to ensure a sufficient amount of nucleic acids. For STS patients, standard of care requires that biopsy samples are stored frozen. In the absence of such a stored material, fresh tumour samples need to be obtained before any specific treatment. WES and RNASeq require two fresh frozen samples for DNA and RNA extraction. The tumour cellularity—that is, the percentage of tumour cells relative to stromal nuclei, normal mucosal or soft tissue content, infiltrating lymphocytes and necrotic areas—of both samples has to be equal to or higher than 30%, in order to avoid the non-detection of rare mutations taking in account the depth of the sequencing (120X). Percentages of tumour cellularity must be noted in the sequencing analysis report. In the case where only one sample fulfilled the tumour cellularity criteria or one specimen only was available, DNA extraction was preferred to RNA extraction. In cases in which the two samples are qualified, the one with the highest tumour cellularity was chosen for RNA extraction.

As most of the mCRC patients in the Multipli study will have their tumour biopsy stored as FFPE blocks, a particular attention was paid to the suitability of FFPE samples as tumour DNA source for WES. FFPE samples are convenient for immunostaining or morphology analyses for diagnosis establishment or prognosis evaluation, and their widespread availability makes them a major valuable resource for cancer studies. However, this type of storage is known to have a significant impact on both DNA quality—namely on the length of the DNA fragments—and the sequencing—with a higher risk of false results due to formalin-linked DNA artefacts. An evaluation was conducted in the broader frame of a CNRGH (National Center of Human Genomics Research) study aiming to compare the performance of three FFPE DNA extraction kits on whole exome sequencing data from FFPE samples.^16^ The evaluation was conducted on samples from five mCRC patients: fresh frozen (FF) samples were taken as gold standard and comparisons were made on the basis of DNA and sequencing quality and variant calling agreement between FF and FFPE samples. Unsurprisingly, the results showed that the FFPE storage and the extraction protocol type have an impact on the DNA quality and the sequencing metrics in terms of rates of PCR duplicates, read length, coverage and calling agreement between FF and FFPE samples *(* *online supplementary data section* *“FFPE samples suitability for NGS“ subsection, table SDI)*. Nevertheless, results showed that FFPE samples could be used in a high-throughput sequencing approach providing the use of a correct DNA FFPE extraction protocol.

### Importance of the FFPE DNA extraction protocol validation step

In accordance with these results, we chose Maxwell protocols for FFPE DNA extraction in the Multipli WES/RNASeq Platforms Workflow Study. Unexpectedly, one of the two INCa-labelled molecular genetics centres involved in the study encountered a FFPE DNA extraction failure resulting in low (<2) or limit (2.4 to 3.0) DNA Integrity Numbers (DINs). The quality of the sequences obtained from these DNAs was not sufficient for any interpretation such as CNV (Copy Number Variants) profile. In contrast, no problems were recorded with the FFPE samples from the other molecular genetics centre. Further enquiries revealed that the techniques used on the two molecular genetics centres were different as to the stock material (core vs slide), the DNA extraction kits. The outcome showed that the use of the Maxwell RSC DNA FFPE AS1450 kit with the Maxwell RSC instrument was responsible for the low DNA extraction quality *(* *online supplementary data section* *“Importance of the FFPE DNA extraction protocol“ subsection, figures SD3 and SD4)*. This barrier was overcome by using another extraction kit with the same instrument. Such troubleshooting underscores the need for the molecular genetics centres and future platforms to systematically assess the effectiveness of the combination of the nucleic acids extraction kit with the instrument used before launching any high-throughput sequencing task.

### Minimal RNA integrity number

The following procedures for nucleic acid qualification were validated by the evaluation study. Once purified, nucleic acids were aliquoted in tubes identified with the patient ID and with a specific barcode provided by the sequencing platform CNRGH and quantified on the INCa-labelled molecular genetics centres. Once received at the sequencing platform, sample identity (consistency between patient ID, barcode and requisition form) was controlled and nucleic acids were quantified and their quality checked again. Methods and results for nucleic acids qualification in the Multipli WES/RNASeq Platforms Workflow Study are detailed in the *online supplementary data section* *(“Nucleic acid qualification (quantity and quality) criteria“ subsection, table SDII)*.

Attention was especially paid to RNAs with lower RNA Integrity Number (RIN). Sequencing was successful for all the tumour RNAs (T-RNAs) with a RIN >6, but the library preparation output was lower for T-RNAs with a RIN <7. Sequencing of T-RNAs with a RIN <3 could not be carried out with the protocol Illumina TruSeq stranded messenger RNA selected in the study. Therefore, RNAs with a RIN <5.5 will not be sequenced in the forthcoming Multipli study, while RNAs with a RIN ≥6 will be systematically sequenced. Regarding RNAs with RIN values between 5.5 and 6.0, the decision to perform sequencing will depend on the RNA fragments length profile and will be discussed case-by-case at the platform managers level.

### Sequencing quality metrics

Sequencing quality was monitored during the run, with expected metrics values set as described in the *online supplementary data section* *(“Sequencing quality metrics“ subsection, table SDIII)*. Metrics assessment showed a significant decrease in the percentage on target for three FFPE tumour DNAs (T-DNAs) and a decrease in the sequencing depth for two of them. This discrepancy between the three FFPE T-DNAs and other samples was found again at the level of the T-DNA coverage profile (*online supplementary data section, figure SD5*). Altogether, these results corroborate the well-known effect of FFPE storage conditions on DNA sequencing quality. As discussed above, this barrier can be expected to be solved, at least in part, by the choice of an adequate extraction protocol and instrument combination. However, special attention remains to be paid to DINs and sequencing quality metrics obtained with FFPE samples in the future clinical routine practice.

### Failure to detect expected mutations

Calling of somatic genetic alterations was made on the Multipli Panel of 90 genes directly targeted by the drugs available in the Multipli trials and on the Cancer Gene Census Panel of 616 genes. Germline variations were searched in the 59 ACMG (American College of Medical Genetics and Genomics) recommended genes.^17^ Presence of polymorphisms known to influence drug metabolism (level of evidence 1A and 1B by the Pharmacogenomics Knowledgebase) was also screened.^18^

Previously identified genetic alterations within patients via NGS panels were also detected in the Multipli WES/RNASeq Platforms Workflow Study, with the exception of mutations in the exon 2 of the KRAS gene in three FFPE T-DNAs. The hypothesis of an intratumourous heterogeneity was ruled out, as these KRAS mutations were detected when using the NGS Colon and Lung Cancer panel on the same FFPE T-DNA. A first analysis showed that, in the three FFPE samples, the coverage depth at the KRAS exon 2 level was lower than the average T-DNA coverage depth (online supplementary data section “bioinformatics and mutations non-detection“ subsection, figure SD6A). **This decrease could not be solely explained by a limited extraction efficiency linked to a low quality of FFPE DNAs, as other FFPE DNAs samples exhibited correct** KRAS exon 2 coverage (online supplementary data section, figure SD6B). The absence of KRAS exon 2 mutation detection appears to be related to a focal exome capture weakness highlighted by FFPE extraction conditions. We thus explored the coverage of the 90 genes of the Multipli panel and confirmed that indeed several exons were never covered. This troubleshooting underscores the crucial importance to report exonic coverage values before any biological interpretation is made. In the Multipli study, an exon by exon coverage graph for each patient will be systematically presented in the homepage of the genVarXplorer tool for final biological report and discussion within the MTB (online supplementary data section, figure SD7).

### Time required for biological interpretation

Data sequencing interpretation was carried out by the biologists of the INCa-labelled molecular genetics centres directly onto genVarXplorer. The interpretation process referred to INCa molecular genetics centre quality assurance programme^19^ (online supplementary data section “biological interpretation“ subsection, figure SD8). The Multipli WES/RNASeq Platforms Workflow Study highlighted the time-consuming aspect of the biological interpretation of sequencing data, related in priority to the high number of genes that needed to be analysed in a cancer patient care setting. The average biological interpretation time was estimated to be of 1.5 to 2 hours, and even more (up to 6 hours) in a microsatellite instability context. This makes this step one of the most critical in the future cancer care pathways that are based on high-throughput DNA sequencing. These estimations highlight the need to develop artificial intelligence tools to help biologists accelerate efficiently the sequencing data interpretation.

### Organisation of molecular tumour boards

Patient tumour profiles were discussed within a weekly MTB meeting. MTB meetings were common to the two diseases (sarcoma and colorectal cancer). The MTB was comprised of the sarcoma and colorectal cancer referring oncologists, one coordinating molecular biologist from an INCa-labelled molecular genetics centre, sarcoma and colorectal cancer expert pathologists from a INCa-labelled molecular genetics centre, the Multipli biology project manager as the MTB session rapporteur, one bioinformatics scientist from the Institut Bergonié bioinformatics platform and the investigators whose patients files were discussed. In the Multipli study, an oncogeneticist will be asked to attend the committee if needed.

As Multipli will be a multicentric study, and Multipli-assigned genomics centres are located in the south and north of France, MTB meetings require to be held via web conferencing and the data accessible to all via a dedicated interface, genVarXplorer. The list of variants previously annotated by the biologists and useful clinicopathological information were shared during MTB via a secured access to the genVarXplorer interface for all the participants. This allowed the selection of annotated variants and MTB conclusions to be collective. In the Multipli study, the MTB will therefore consensually designate the more relevant actionable alteration to be targeted by one of the available drugs. Of note, in the small number of patients tested in this feasibility study, no further molecular abnormalities targets of current available drugs were identified.

### Molecular Tumour Board report

**Figure 4:**
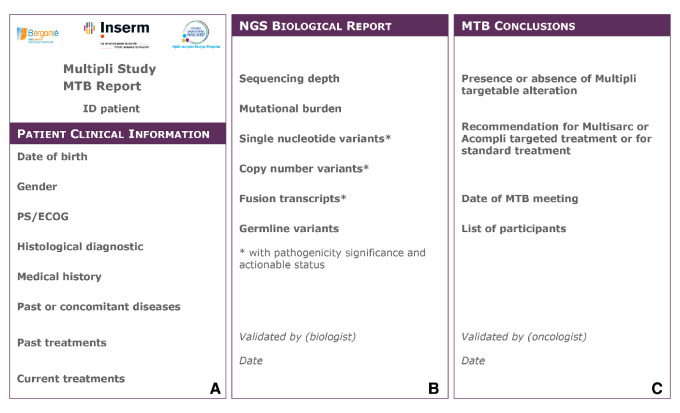
The Multipli Molecular Tumour Board (MTB) Report. For each Single Nucleotide Variant, gene access number (NM), Human Genome Variation Society (HGVS) nomenclature, level of pathogenicity and actionability and eventual association with a loss of heterozygosity have to be reported. For each Copy Number Variant, segment size, copy numbers, level of pathogenicity and actionability have to be reported. For each Fusion Transcript, nomenclature, breakpoint, consequence on open reading frame, level of pathogenicity and actionability have to be reported. For each Germline Variant reported, gene access number (NM), HGVS nomenclature, zygosity and level of pathogenicity have to be detailed. Drug toxicity associated variants with a 1A or 1B level of evidence (based on PharmGKB data) are reported. ID patient, patient identification number;MTB, Molecular Tumour Board; NGS, next-generation sequencing; PS/ECOG:Performance Status on the ECOG (Eastern Cooperative Oncology Group) scale.

The final MTB report was composed of three successive parts corresponding respectively to (a) the patient clinicopathological background, (b) the sequencing biological interpretation itself and (c) the clinical MTB conclusions (figure 4). A biologist and an oncologist participating in the MTB meeting validated the MTB report, which was then notified to the patient’s physician and the tumour board.

## Conclusion

High-throughput sequencing techniques are expected to be implemented in the clinical cancer care routine. A growing number of studies explore the conditions under which the implementation of high throughput WES/RNASeq in the clinics will be valid in terms of feasibility, results accuracy, clinical benefit, and so on.^5–12^ In a routine care practice, cancer patients who would be referred for genomic analysis and the platforms for these studies will lean on coordinated multicentre structures. Assessing the feasibility of this implementation in the French clinical pathway is the primary objective of the Multipli study, as one of the four pilot projects of the national FGM 2025 Plan. To prepare for such a national organisation, the Multipli WES/RNASeq Platforms Workflow Study provide guidelines and SoP support for the forthcoming development of genomic analysis in the cancer patient care.

**Figure 5:**
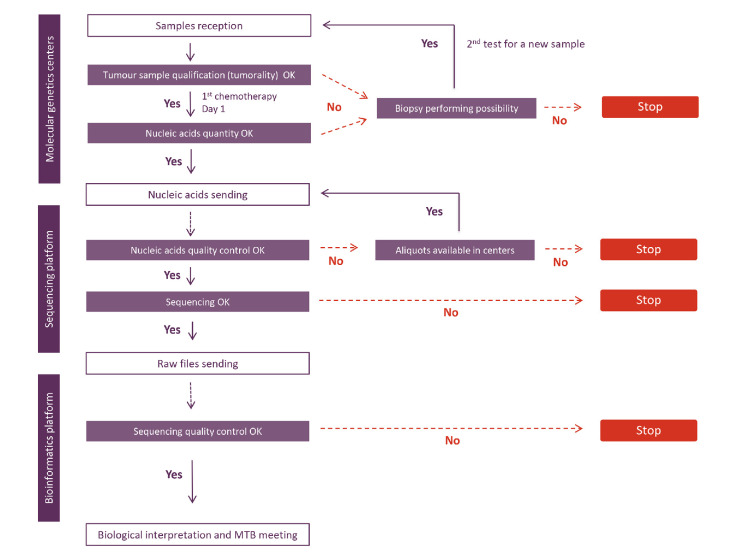
The decision-making tree for Multipli study continuation or discontinuation. MTB, Molecular Tumour Board; SOP, Standard Operating Procedures.

The study shows the feasibility of tumour genomic analysis by WES/RNASeq in a time frame compatible with the current patient care, that is, 7 weeks. The delays observed allowed us to identify steps which could be redone in the time frame and those which could not. We detailed in the online supplementary data section the different steps, foreseen barriers and methods to provide alternative solutions. Several barriers have been identified and mastered in the course of the genomic workflow. Many of them were a consequence of the FFPE processing of biopsies. Teams must be aware of the attention to be paid when using FFPE samples for sequencing. A decision-making tree was thus defined (figure 5), which will help forthcoming Multipli teams to determine study continuation or discontinuation for inadequate quantity or quality samples.

Biological interpretation is a crucial step in the analysis. We benefitted from a multiplatform molecular board to synergise expertise and stress the importance of further developments to automate the MTB report filling, such as the direct importation of clinical information from the clinical electronic case report form before MTB and the automatic importation of biological information selected during MTB from the genVarXplorer bioinformatic tool.

Finally, above and beyond the technical barriers that we have identified, and in order to attain substantial gain of time and efficiency necessary in patient care pathway, such a project highlights the need of well trained and dedicated personnel for the coordination of the medical and scientific teams engaged in the next generation of clinical genomic analysis.

Aware that the steps and recommendations presented here are deemed to progress over time, we wish to share our experience in setting up a WES/RNASeq Platforms Workflow in a cancer patient care pathway. The procedures identified should provide a canvas for different clinical centres and different pathologies, molecular and genomic platforms wishing to launch in this endeavour.
