# Supplementary material for: Feasibility of high-throughput sequencing in clinical routine cancer care: lessons from the cancer pilot project of the France Genomic Medicine 2025 plan
Source: ESMO Open. 2020 Jul 26;5(4):e000744. doi: 10.1136/esmoopen-2020-000744 (PMC7383956; doi:10.1136/esmoopen-2020-000744)
Supplement: Supplementary data [file esmoopen-2020-000744supp001.pdf]

Feasibility of High Throughput Sequencing in Clinical Routine Cancer Care: Lessons from the Cancer Pilot Project of the France Genomic Medicine 2025 Plan

THE FGM 2025 MULTIPLI WORKFLOW STUDY GROUP.

Céline Auzanneau[1,2], Delphine Bacq[3], Carine Bellera[4], Hélène Blons[5], Anne Boland[3], Marlène Boucheix[1], Aurélien Bourdon[2,7], Emmanuelle Chollet[8], Christine Chomienne[8,9], Jean-François Deleuze[3,10], Christelle Delmas[11], Derek Dinart[4], Hélène Espérou[11], Flore Geillon[12], Damien Geneste[2,7], Antoine Italiano[2,13], Delphine Jean[4], Emmanuel Khalifa[1,2], Yec'han Laizet[2,7], Pierre Laurent-Puig[5,6], Franck Lethimonnier[14], Claire Lévy-Marchal[11], Carlo Lucchesi[2,7], Carine Malle[11], Pierre Mancini[2,7], Simone Mathoulin-Pélissier[4], Vincent Meyer[3], Marie-Ange Palomares[3], Géraldine Perkins[5,6], Sabrina Sellan-Albert[3], Isabelle Soubeyran[1,2], Cédric Wallet[4]

Authors affiliation

1. Unité de pathologie moléculaire, Institut Bergonié, Comprehensive Cancer Centre, Bordeaux, France
2. Inserm U1218, Institut Bergonié, Comprehensive Cancer Centre, Bordeaux, France
3. Centre national de recherche en génomique humaine (CNRGH), Institut de biologie François-Jacob, CEA, Université Paris-Saclay, Évry, France
4. CIC-EC 1401/EUCLID - Institut de santé publique, d'épidémiologie et de développement (Isped), Université de Bordeaux, France
5. Inserm U1147, Centre universitaire des Saints-Pères, Paris, France
6. Génétique médicale et clinique, Hôpital européen Georges-Pompidou, Paris, France
7. Unité de bioinformatique, Institut Bergonié, Bordeaux, France
8. Alliance nationale pour les sciences de la vie et de la santé (Aviesan), Institut thématique multiorganisme Cancer, Paris, France
9. Institut national du Cancer (INCa), Boulogne-Billancourt, France
10. Centre de recherche, d'innovation et d'expertise (CREFIX), US39, CEA, Evry, France
11. Inserm, Institut de santé publique, Pôle recherche clinique, Paris, France
12. Fédération française de cancérologie digestive (FFCD), Dijon, France
13. Early Phase Trials and Sarcoma Units, Institut Bergonié, Comprehensive Cancer Centre, Bordeaux, France
14. Alliance nationale pour les sciences de la vie et de la santé (Aviesan), Institut thématique multiorganisme Technologies pour la santé, Paris, France

Corresponding author

Christine Chomienne

Alliance nationale pour les sciences de la vie et de la santé (Aviesan), Institut thématique multiorganisme Cancer, Paris, France and Institut national du Cancer (INCa), Boulogne-Billancourt, France – email: christine.chomienne@inserm.fr

Auzanneau C, *et al. ESMO Open* 2020; 5:e000744. doi: 10.1136/esmoopen-2020-000744

Supplementary Data

1. THE FUTURE FRENCH ROUTINE CANCER CARE PATHWAY

- Figure SD1: Schematic representation of the future French routine cancer care pathway

2. THE FRANCE GENOMIC MEDICINE 2025 PLAN

- Figure SD2 : How to make genomic medicine accessible to all patients? Objectives and measures of the FGM 2025 plan, with a special highlight on the feasibility of NGS sequencing in clinical routine (pilot projects of the measure #5)

3. METHOD OVERVIEW OF THE MULTIPLI WES/RNASEQ PLATFORMS WORKFLOW STUDY

4. METHOD STUDY AND RESULTS FOR FFPE SAMPLE SUITABILITY FOR NGS

- Table SDI: Impact of FFPE storage and FFPE DNA extraction on DNA and sequencing quality
- Figure SD3: The Maxwell®RSC DNA FFPE with the Maxwell®RSC instrument does not allow to obtain sufficiently high DINs
- Figure SD4: The Custom kit used with the Maxwell®RSC instrument is relevant for FFPE DNA extraction and will be used in Multipli study

5. NUCLEIC ACID QUALIFICATION (QUANTITY AND QUALITY) CRITERIA

- Table SDII: DNAs and RNAs quality control results
- Table SDIII: Expected sequencing quality metrics
- Figure SD5: Low DIN FFPE DNAs showed affected sequencing control metrics

7. BIOINFORMATICS AND MUTATIONS NON-DETECTION

- Figure SD6: Coverage depth of A) The KRAS exon 2 gene as compared to T-DNA (Tumoral DNA) and B) All study samples at the KRAS gene level
- Figure SD7: Multipli genes panel coverage report on the genVarXplorer tool homepage

8. BIOLOGICAL INTERPRETATION

- Figure SD8: The Multipli WES/RNASeq biological interpretation process according to INCa recommendations.

1. The future French routine cancer care pathway

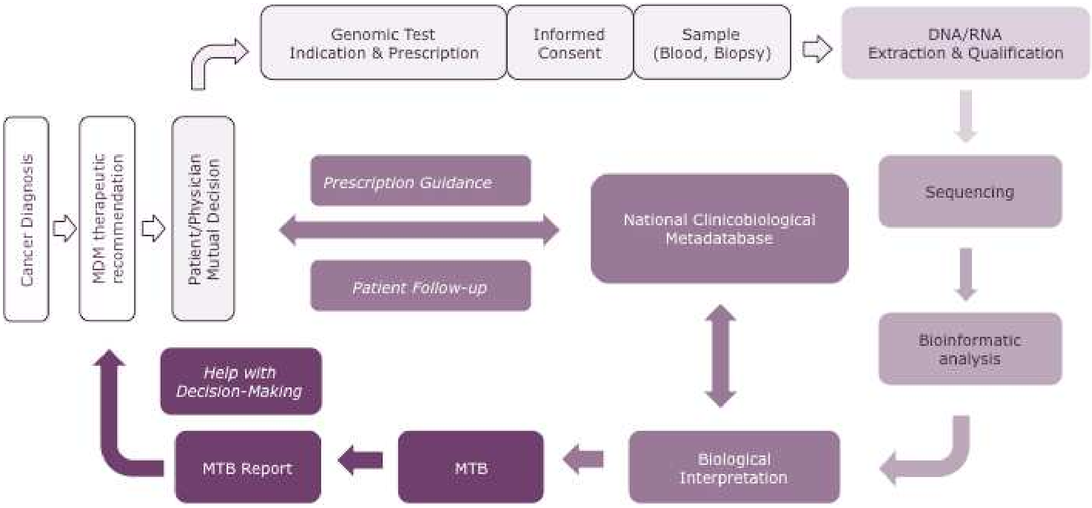

Figure SD1: Schematic representation of the future French routine cancer care pathway
MDM: MultiDisciplinary meeting; MTB: Molecular Tumour Board

2. The France Genomic Medicine 2025 plan

The France Genomic Medicine 2025 plan launched in 2016 aims to construct a French medical and industrial system to introduce precision medicine into the routine care pathway.

Figure SD2: How to make genomic medicine accessible to all patients: Objectives and measures of the

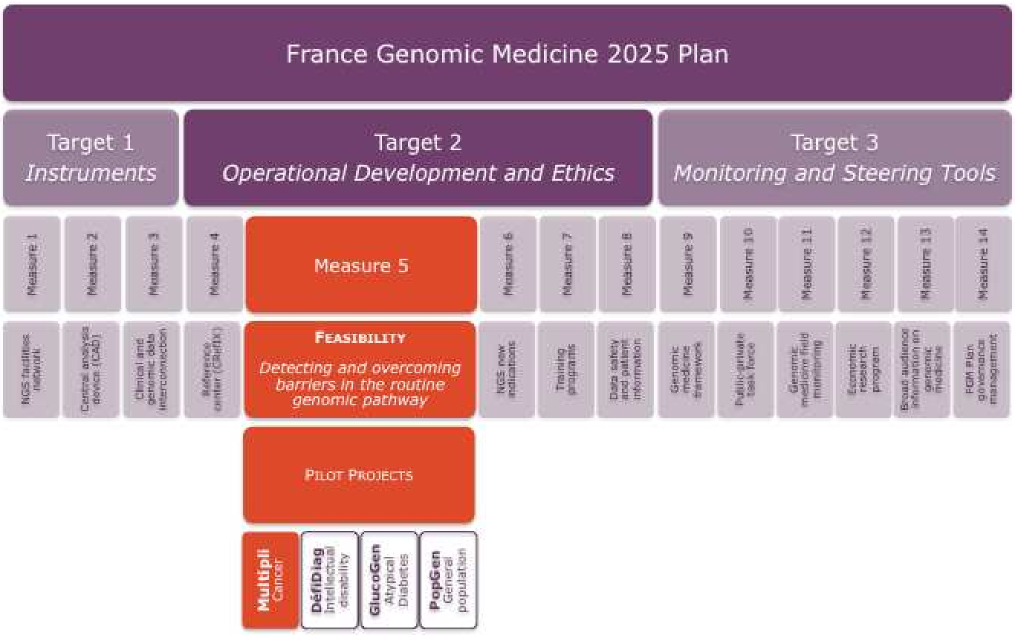

FGM 2025 plan, with a special highlight on the feasibility of NGS sequencing in clinical routine (pilot projects of the measure 5)

The plan is articulated around 3 main objectives – Establishing instruments for a genomic care pathway; Ensuring operational development of the system in an ethical framework; Establishing the monitoring and steering tools throughout the implementation of the plan - and 14 measures. Among those, measure 5 aims to detect and overcome the technological, clinical and regulatory obstacles encountered along the genomic care pathway with respect to three broad groups of diseases and to a

Auzanneau C, *et al. ESMO Open* 2020; 5:e000744. doi: 10.1136/esmoopen-2020-000744

sample of the general population. Four pilot projects have been designed for this purpose, including the Multipli project for cancer disease.

The Multipli study will be a large, multicenter study carried out in around 2,400 patients. It will encompass two innovative trials driven in patients suffering from two different types of cancer: a soft-tissue sarcoma (STS, Multisarc trial), which is a rare but severe cancer, or a colorectal carcinoma (CRC, Acompli trial), which is a more frequent cancer with a better prognosis. Multisarc and Acompli trials will have as primary objective to assess the feasibility of WES/RNASeq of tumor genomes in the time frame required for the 1st line patient treatment. According to the efficiency of the 1st line induction therapy, WES/RNASeq results may be useful to the patient by guiding a targeted therapy provided by the Multipli clinical trials. As secondary objectives, Multisarc and Acompli trials will assess the benefit of such NGS-guided targeted treatments on disease non-progression and patient's survival compared to standard therapy.

3. Method overview of the Multipli WES/RNASeq Platforms Workflow Study

Twenty-four (24) blood samples and 23 tumour samples were collected, one STS (Soft Tissue Sarcoma) biopsy having been scheduled out of the time of the study. Two (2) mCRC (metastatic ColoRectal Cancer) tumour samples were not qualified (tissue necrosis or tumour cellularity close to zero). Thus, 24 blood samples and 21 tumour samples were qualified for nucleic acid extraction. Among those 21 tumour samples, 11 were Fresh Frozen (FF) material and 10 were Formalin-Fixed Paraffin-Embedded (FFPE) material. DNA extraction was possible for all blood and all FFPE samples. RNA extraction was performed from FF samples only. Two specimens were required for FF samples, one for DNA extraction, the other for RNA extraction. For 3 patients, only one FF specimen was qualified, resulting in the loss of RNA or DNA extraction possibility. Altogether, 20 tumoral DNA (T-DNAs), 9 tumoral RNAs (T-RNAs) and 24 constitutional DNAs (C-DNAs) were obtained after extraction (3 C-DNAs were not taken into account because of the absence of tumoral paired specimen). The 21 C-DNAs had a high DNA Integrity Number (DIN > 7) and were therefore qualified. FF T-DNAs were all but one (DIN = 2.6) of good quality (DIN > 6.5), while FFPE T-DNAs had limit (2.4 < DIN < 3, n=5) or low (DIN < 2, n=5) DINs. All the 20 T-DNAs were still taken into account for the next step. Quality heterogeneity was also observed for the 9 T-RNAs (RIN > 6, n = 6 ; RIN < 3, n = 3). Despite a very low quality (RIN = 1.6), one of the 3 low RIN T-RNAs was still taken into account because of the absence of paired T-DNA, but no library could be obtained. One C-DNA was thus excluded because of the absence of tumoral paired specimen. Twenty (20) C-DNAs, 20 T-DNAs and 6 T-RNAs were sequenced, representing data sets (C-DNA + T-DNA ± T-RNA) for 20 patients. Finally, twenty (20) patient sets of sequencing data were submitted to bioinformatic analysis and biological interpretation. Files from 20 patients (7 STS and 13 mCRC) were discussed during one of the six Molecular tumour board (MTB) meetings of the study. All but one steps were performed within expected times. The slightly longer duration of the sequencing step (22 vs 21 calendar days expected) was an artifact due to the organisation adopted for the study, which stipulated that a sequencing cycle would be launched when a minimal number of samples were available. In normal conditions, the duration of this step is actually estimated to be strictly less than 21 calendar days.

4. Method study and results for FFPE sample suitability for NGS

The experiment was carried out on samples from 5 mCRC (metastatic ColoRectal Cancer) patients treated in the Hôpital européen Georges-Pompidou (HEGP) digestive oncology department. Tumor DNA was extracted from Fresh Frozen (FF) sample with the Maxwell® RSC Tissue DNA Kit (TM) protocol. For Formalin-Fixed Paraffin-Embedded (FFPE) samples, 3 protocols were assessed: the GeneRead DNA FFPE Kit (TG), the QIAamp DNA Mini Kit (TQ) and the Maxwell® 16 DNA FFPE Kit AS1135 (TX). Three (3) genes panels - Multipli (70 genes at the time of the experiment), Multipli Extended (618 genes) and Whole Exome (≈ 18,000 genes) - were targeted by the sequencing analysis. FFPE/FF agreement was defined as the percentage of variants detected in FF samples which are also detected in at least one FFPE sample, either all FFPE DNA extraction protocols taken together (Overall FFPE) or according to the FFPE DNA extraction protocol. DNA sequencing from FFPE samples showed a higher rate of PCR duplicates (X3 on average, and almost X4 when GeneRead DNA FFPE kit was used) and a shorter reads length (20% less on average) than DNA from FF samples. DNA coverage values were less affected, except when the GeneRead DNA FFPE kit was used (a 45%

coverage was obtained, vs 60% for FF DNA). Similarly, storage and extraction methods have an impact on the FFPE/FF variant calling agreement, both overall (agreement varying between 69% and 74% depending on the gene panel considered) and even more when considering the GeneRead DNA FFPE kit extraction protocol alone (37% of FFPE/FF agreement on average, vs 72% on average for the overall FFPE). This was true whatever the gene panel targeted, i.e., Multipli Extended or Whole Exome (no conclusion could be drawn for the Multipli gene panel because of a too small number of detected variants in either FF or FFPE samples).

Table SDI: Impact of FFPE storage and FFPE DNA extraction on DNA and sequencing quality

		Protocols			
		FF	FFPE		
**DNA and sequencing quality indicators**		TM	TG	TQ	TX
PCR duplicates (%)		11	43	20	28
Coverage per base at a 30X depth (%)		≈ 60	45	50	≈ 60
Reads sequence length (bp)		75	60	60	60
**Variant calling: FFPE/FF agreement (%)**					
Overall FFPE	*Multipli Extended*	-		≈ 74	
	Whole Exome	-		≈ 69	
According to the FFPE protocol	*Multipli Extended*	-	28	≈ 67	≈ 72
	Whole Exome	-	45	≈ 70	≈ 59

Maxwell protocols were chosen for FFPE DNA extraction: Maxwell®16 FFPE Plus LEV DNA purification AS1135 (LEV Kit) with the Maxwell® 16 instrument (Molecular Genetics Center#1, Center#1) and kit Maxwell® RSC DNA FFPE AS1450 (RSC Kit) with the Maxwell® RSC instrument (Center#2). Unexpectedly, 5 of the 8 FFPE samples from the Center#2 encountered DNA extraction failure with resulting low (< 2) DNA Integrity Numbers (DINs), while the remaining 3 DNAs exhibited limit but expected from FFPE samples DIN values (2.4 < DIN < 3.0). In contrast, the DNA quality of the two FFPE DNA from Center#1 was of sufficient quality for sequencing interpretation. Comparison of the techniques used on the two centers led us to make two assumptions explaining this DNA extraction failure, involving either the stock material type - core (Center#2) vs slide (Center#1) - or the DNA extraction kit type - RSC Kit (Center#2) vs LEV Kit (Center#1). An additional work was carried out on 4 of the 8 FFPE DNA in extraction failure to conclude with these 2 hypotheses (*Figure SD3*): 25µm slides were performed by the Center#2 on each one of the 4 FFPE blocks (1st hypothesis testing). DNA extraction was performed on the 2 set of 4 slides either with the RSC kit with the Maxwell®RSC instrument (Center#2) or with the LEV Kit with the Maxwell®16 instrument (Center#1) (2nd hypothesis testing). DINs obtained according to the stock material type (core vs slide) did not exhibit significant variations (1.3-2.8 vs 1.7-2,4, respectively). However, a significant improve in DINs (4.5-4.8 vs 1.7-2.4) was observed when the LEV kit with the Maxwell®16 instrument was used instead of the RSC kit with the Maxwell® RSC instrument. Results validated the 2nd hypothesis: the Maxwell® RSC DNA FFPE AS1450 kit with the Maxwell® RSC instrument does not allow to obtain DNA of sufficient quality for NGS, leading us to exclude this combination from the Multipli study.

Auzanneau C, *et al. ESMO Open* 2020; 5:e000744. doi: 10.1136/esmoopen-2020-000744

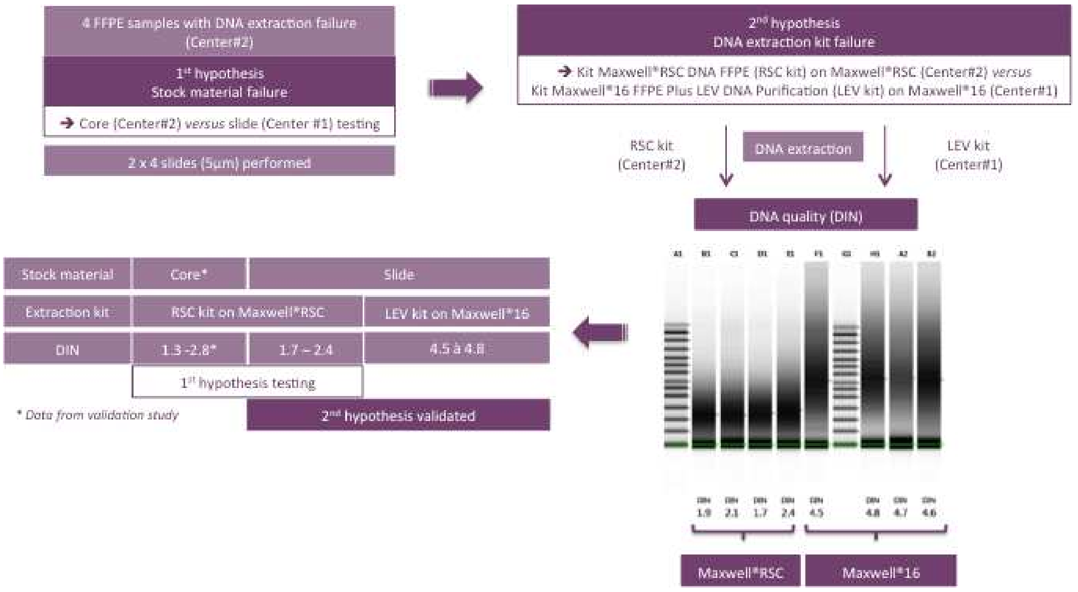

Figure SD3: The Maxwell®RSC DNA FFPE with the Maxwell®RSC instrument does not allow to obtain sufficiently high DINs.

A Maxwell® RSC FFPE Plus DNA Purification Kit Custom (Custom kit) with chemical properties similar to those of the LEV kit but compatible with the Maxwell® RSC instrument was provided by the supplier and assessed on 3 FFPE samples with former DNA extraction failure (*Figure SD4*).

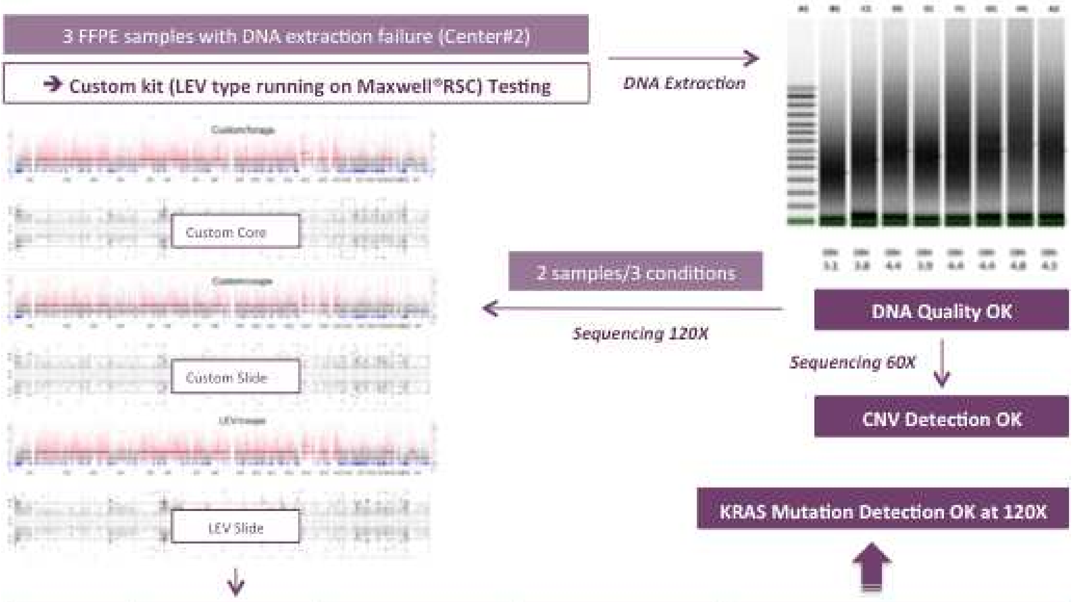

Sample	LEV Slide		Custom Slide		Custom Core		KRAS Variant
Coverage	60X	120X	60X	120X	60X	120X	
#1	13/38	24/81	4/43	15/72	2/24	13/68	NM 033360:exon2:c.38G>A:p.G13D
#2	5/34	14/93	2/41	10/66	5/29	14/68	NM 033360:exon2:c.35G>T:p.G12V

Figure SD4: The Custom kit used with the Maxwell®RSC instrument is relevant for FFPE DNA extraction and will be used in Multipli study

The Custom kit allowed DNA extraction with an efficiency close to that expected (DINs 3.1-4.8). Similarly, CNV analysis results were close to those obtained with the LEV kit. However, results

regarding the mutation detection were less clear because of the too low sequencing coverage (60X) used at first. This was particularly true for mutations with low allele frequency such as KRAS mutations. Thus, 2 of the 3 previous FFPE samples with known low frequency KRAS mutations were sequenced at a 120X coverage according to 3 conditions of extraction kit and stock material: LEV/Slide, Custom/Slide and Custom/Core. Results showed that increasing the sequencing coverage from 60X to 120X (which is the value recommended for the Multipli study) was able to overcome the KRAS non-detection problem whatever the condition used.

5. NUCLEIC ACID QUALIFICATION (QUANTITY AND QUALITY) CRITERIA

Methods: Briefly, the DNA quantification was performed in duplicate using a broad range or high sensitivity Quant-ITdsDNA assay kit according to the sample DNA concentration estimated at the INCa-labeled molecular genetics center. The minimum amount of DNA required for NGS testing was set at a value equal to or higher than 500 ng per sample at a 10-30 ng/µL concentration. This amount allowed the CNRGH to perform the quality control and the exome capture with the set required quantity of 200 ng and, if necessary, another exome capture. RNA quantification was performed in duplicate using a NanoDrop device. Amount and concentration values required for RNA samples were equivalent to those described above for DNA samples (200 ng of RNA are required for the mRNASeq library preparation). A DNA or a RNA amount lower than 200 ng and a DNA or a RNA concentration lower than 4 ng/µL constituted an exclusion criteria. DNA quality was assessed by the DNA Integrity Number (DIN), which was determined using the migration of a small amount (20 ng) of DNA sample on Tape Station 4200 (Agilent Technologies). A DNA with a DIN > 7 is usually considered to be of good quality and suitable for NGS, while a DIN < 5 usually reflects a low quality DNA. RNA quality was assessed by the RNA Integrity Number (RIN), which was determined using the migration of a small amount (20-50 ng) of RNA sample on a Bioanalyzer 2100 chip (Agilent Technologies). A RNA with a RIN > 7 is usually considered to be of good quality, while a RNA with a RIN < 5 is considered to be of low quality

Results: Regarding quantification, 2 of the 21 constitutional DNAs (C-DNAs) and 2 of the 20 tumour DNA (T-DNAs) were received at the CNRGH sequencing platform in a limited quantity (< 400 ng) which would have compromised a second sequencing if necessary. All other samples were in sufficient DNA quantity (> 500 ng). Among tumour RNAs (T-RNAs), 6 were in sufficient quantity, 1 was in intermediate quantity and 2 were in too low quantity (< 200 ng). However, in the context of the evaluation study, these too low T-RNA quantity samples were not excluded. Regarding quality of nucleic acids, all C-DNAs were qualified (DIN > 7) while quality of T-DNAs extracted from FFPE samples varied a lot: 6 T-DNAs were limit (2.4 < DIN < 3) and 5 T-DNAs were of low quality (DIN < 2). For FF samples, 9 T-DNAs were qualified (DIN > 6.5) and 1 T-DNA was unexpectedly of low quality (DIN = 2.6). There was a RIN heterogeneity for the 9 tumor RNAs, with 6 of them of good quality (RIN > 7), 2 of them of limited quality (6.0 < RIN < 6.5) and 3 of them of low quality (RIN < 3). In parallel to nucleic acid quantity and quality assessments, a PCR amplification test was performed in DNA samples to assess the presence of inhibitors, known to have an impact on exome capture efficiency. However, the presence of PCR inhibitors did not constitute an exclusion criteria of the sample.

Table SDII: DNAs and RNAs quality control results

	Quantity			Quality		
	OK<br>> 500 ng	Limit<br>< 400 ng	Low<br>< 200 ng	OK	Limit	Low
C-DNA	19	2	0	21<br>DIN > 7	0	0
T-DNA	18	2	0	9<br>DIN > 6.5	6<br>2.4 < DIN < 3.0	5<br>DIN < 2
T-RNA	6	1	2	4<br>RIN > 7	2<br>6.0 < RIN < 6.5	3<br>RIN < 3

6. SEQUENCING QUALITY METRICS

<u>Methods</u>: C-DNAs and T-DNAs were captured and library preparation and exome enrichment performed at the CNRGH sequencing platform. Exome enriched libraries were sequenced and samples were multiplexed in order to reach a mean coverage of at least 60 X for C-DNA samples and 120X for T-DNA samples. Sequence quality parameters were monitored during the sequencing run, with expected metrics values set as described below (*Table SDIII*).

Table SDIII: Expected sequencing quality metrics

Metrics	C-DNA	T-DNA
Duplicates (%)	< 30 %	
Sequencing depth (%)	> 30 X for 80 % NT	> 60 X for 80 % NT and > 120 X for 50 % NT
Percentage on target (%)	> 75 %	

<u>Results</u>**:** Sequences were obtained for all 41 DNAs, including those with low DINs. Metric assessment showed a significant decrease in the percentage on target for 3 FFPE T-DNAs (V3, V4 and V5) and a decrease in the sequencing depth for 2 of them (V4 and V6). The discrepancy between these 3 FFPE T-DNAs and other samples was also found at the level of the T-DNA coverage profile (*Figure SD5*). Altogether, these results corroborate the well-known effect of FFPE storage on the DNA sequencing quality. For RNAs, libraries obtained were checked for their quality prior to the sequencing. The coverage of the sequencing was estimated through the number of reads produced, with a set value of 90 million reads per sample.

Figure SD5: Low DIN FFPE DNAs showed affected sequencing control metrics

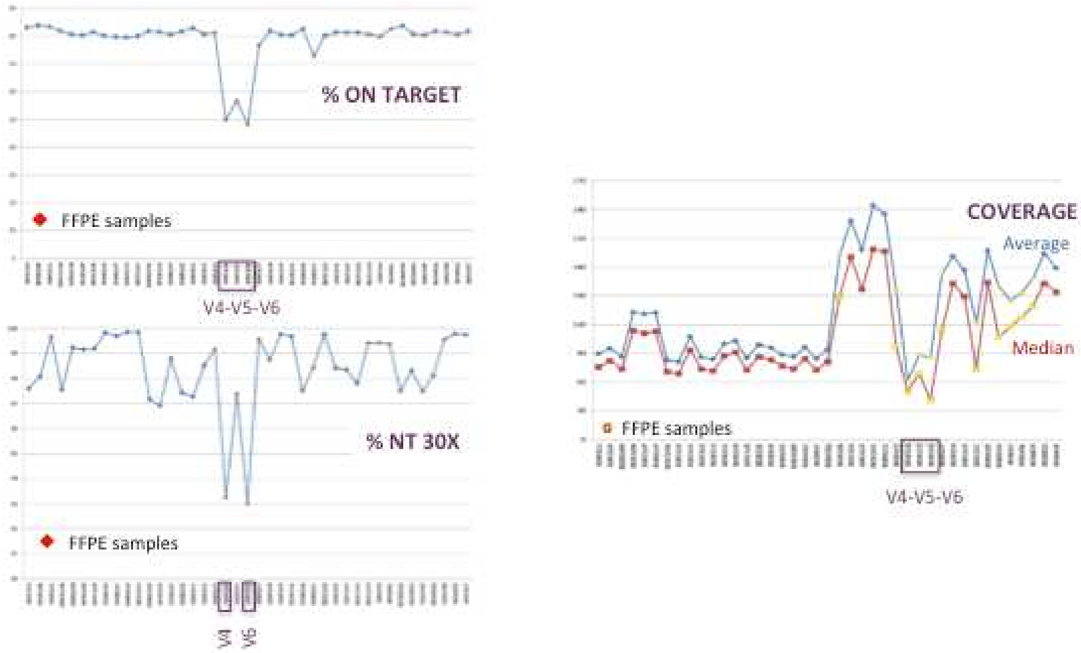

7. BIOINFORMATICS AND NON-DETECTION OF MUTATIONS

<u>Methods</u>: The standard bioinformatic analysis of sequencing data was based on the Illumina generated FastQ file for each sample (DNA and RNA). These raw files were made available to the

Institut Bergonié bioinformatics platform via a CNRGH secured server. Primary analyses - cleaning and alignment of sequences - and secondary analyses – detection of Single nucleotide variants (SNV), InDel, Copy number variants (CNV), fusion transcripts and determination of the tumor mutational burden - were made and the results reported in the genVarXplorer (GVX) tool developed and adapted to the Multipli study by the Institut Bergonié bioinformatics platform. GVX is used as a technical support for the biological interpretation. On GVX, filtered data appear prioritized and annotated. They are connected to databases available online, such as ICGC (International Cancer Genome Consortium) Data Portal, Tumor Portal, Cosmic, OncoKB, etc. Somatic genetic alterations search (T-DNA and RNA) was made on the Multipli Panel of 90 genes, direct targets of the drugs available in the Multipli trials, and on the Cancer Gene Census Panel of 616 genes. The analysis also focused on the germline variants (C-DNA) of the 59 ACMG (American College of Medical Genetics and Genomics) genes known or expected to constitute risk factors for several genetic diseases (secondary findings). Polymorphisms linked to drug toxicities with a level of evidence 1A and 1B by the Pharmacogenomics Knowledgebase were also searched on C-DNA.

Results**:** An absence of detection of *KRAS* mutations was observed, which can be explained by a coverage depth decrease at the *KRAS* exon 2 level (*Figure SD6*). For each patient, Multipli genes panel coverage has to be systematically reported on the genVarXplorer tool homepage (*Figure SD7*).

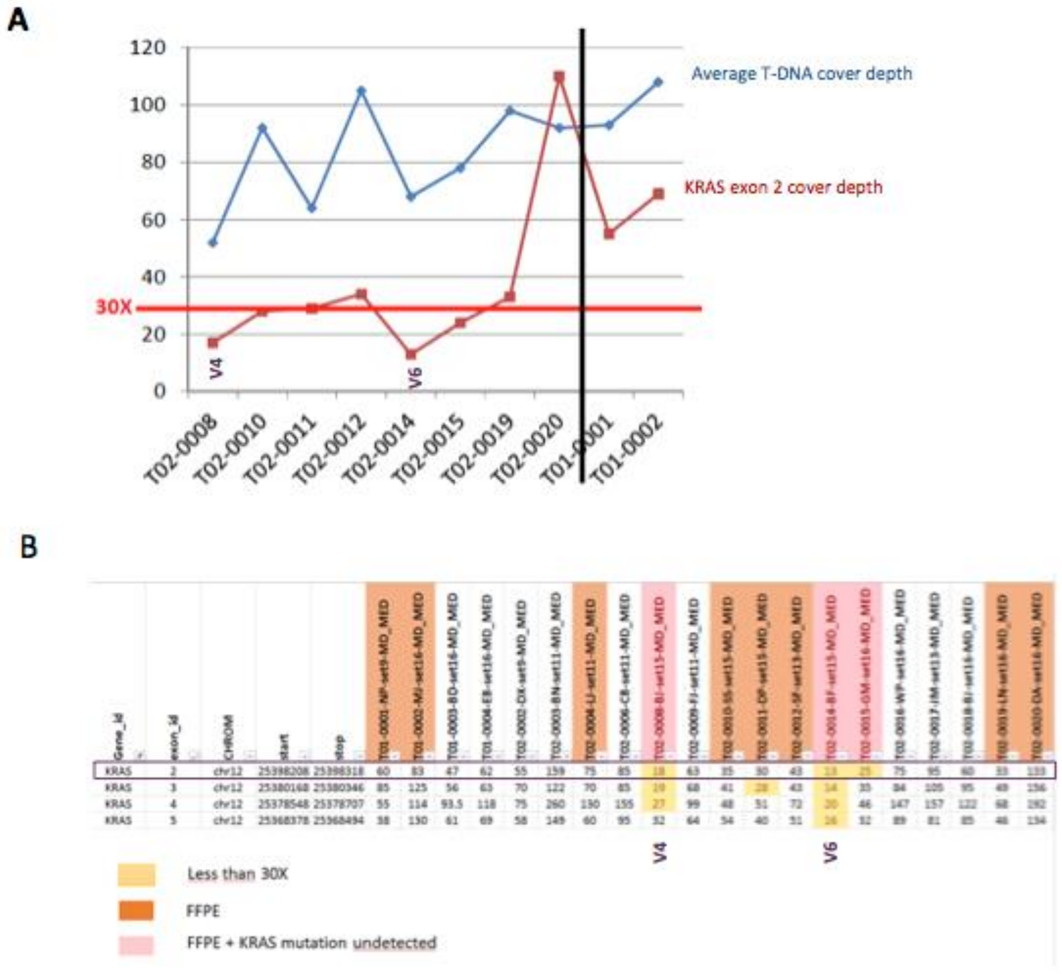

Figure SD6: Coverage depth of A) The KRAS exon 2 gene as compared to T-DNA (Tumoral DNA) and B) All study samples at the KRAS gene level

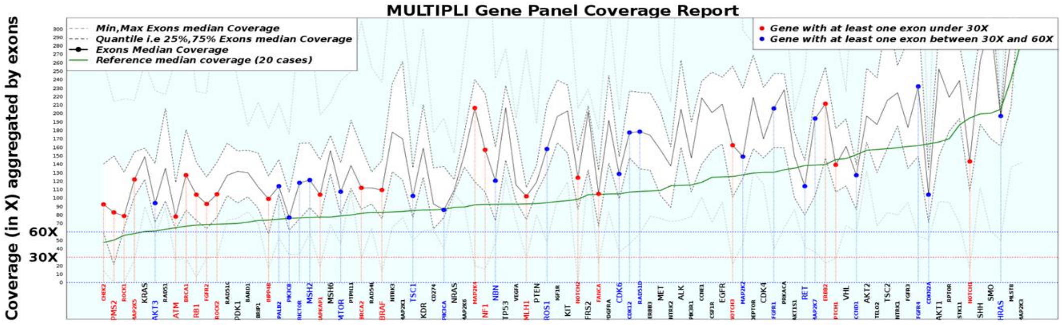

Figure SD7: The Multipli genes panel coverage report on the genVarXplorer tool homepage

8. BIOLOGICAL INTERPRETATION

For each patient, annotated variants were classified on the basis of their pathogenicity, their actionability and their potential targeting by a drug available in the Multipli study. This patient tumor profile - variants classification and comments - was retrievable on the genVarXplorer interface as a first homepage describing sequencing quality and mutational burden with 4 further pages dedicated to somatic SNVs, somatic CNVs, RNA alterations and germline variations. These 5 pages served as a basis for discussion during molecular tumor board (MTB) meetings. In order to guarantee an analytical reproducibility on all patients sequenced during the Multipli study, the annotations and comments relating to each variation will be saved in a specific database, easily consultable through genVarXplorer.

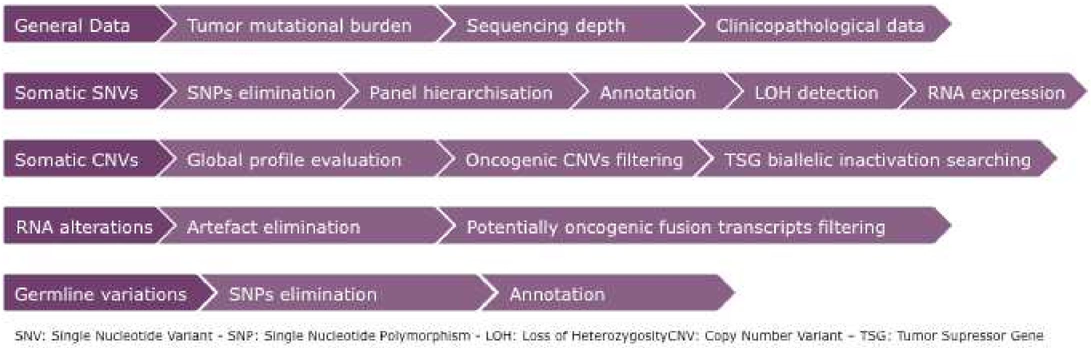

Figure SD8: The Multipli WES/RNASeq biological interpretation process according to INCa recommendations

The analysis focused on Single Nucleotide Variants (SNVs), Insertions/Deletions (InDels), Copy Number Variants (CNVs) and expression of the mutated allele (*Figure SD8*). Regarding SNVs, somatic variations were prioritized by gene panels (Panel hierarchisation) and decreasing allelic fraction in order to optimize the annotation on the most relevant variations. In addition, annotated variants expression in tumoral RNA and association with a Loss Of Heterozygosity (LOH) or gene amplification was taken into account. Regarding CNVs, visualization of the global profile allowed in particular to quantify the degree of recombination and to identify amplified or deleted segments. Bioinformatic filters permitted to prioritize the analysis on relevant CNVs such as oncogene amplifications and Tumor Suppressor Genes (TSG) deletions. For oncogenes, gain-of-function variations (focal amplifications, missense variants, in frame indel, in frame exon splicing mutations) were taken ino account while frameshift mutations, nonsense and synonyms mutations and large deletions were excluded. Inversely, in tumor suppressor genes, loss-of-function variations (mostly variants involved in loss of heterozygosity: stop mutations, known loss-of-function missense variants, and all frameshift variants: indel, splicing mutations, large (intragenic) deletion or insertion) were

taken into account. Regarding RNA alterations, specific filters permitted to optimize the validation on relevant fusion transcripts, such as those for which at least one partner is either an oncogene or a TSG, with sufficient coverage and/or registration in somatic databases. Regarding germline variations located in the 59 ACMG (American College of Medical Genetics and Genomics)-recommended genes, only those with allelic frequencies below 0.5% in all 1000 genome and ExAC (Exome Aggregation Consortium) subpopulations were subjected to annotation and reported in the genVarXplorer analysis tool. Depending on genotype and level of pathogenicity, the Molecular Tumour Board will either request advice of an expert geneticist or refer the patient to a specialized genetic consultation. Presence or absence of polymorphisms known to influence drug metabolism (level of evidence 1A and 1B by the PharmaGKB web site) were also notified
